# Comparative Agreement Analysis of Differences in 1,5-Anhydroglucitol, Glycated Albumin, and Glycated Hemoglobin A1c Levels between Fasting and Postprandial States in Steamed Bread Meal Test

**DOI:** 10.1155/2017/5917293

**Published:** 2017-09-20

**Authors:** Hang Su, Yufei Wang, Xiaojing Ma, Xingxing He, Lingwen Ying, Junling Tang, Lu Dong, Yuqian Bao, Jian Zhou, Weiping Jia

**Affiliations:** Department of Endocrinology and Metabolism, Shanghai Clinical Center for Diabetes, Shanghai Key Laboratory of Diabetes Mellitus, Shanghai Diabetes Institute, Shanghai Jiao Tong University Affiliated Sixth People's Hospital, Shanghai 200233, China

## Abstract

**Background:**

Our previous study indicated that serum 1,5-anhydroglucitol (1,5-AG) levels slightly increased after a glucose load; therefore, this study was conducted to explore short-term changes in 1,5-AG levels after a steamed bread meal test (SBMT) and compare the agreement of 1,5-AG, glycated albumin (GA), and glycated hemoglobin A1c (HbA1c) levels between fasting and postprandial states after an SBMT.

**Methods:**

104 participants were recruited and underwent a 100 g SBMT. Fasting, 30 min, and 120 min of 1,5-AG, GA, and HbA1c were measured.

**Results:**

Levels of 1,5-AG slightly increased from 30 to 120 min after an SBMT (*P* < 0.01), and HbA1c and GA levels showed stability at 30 and 120 min. The Passing-Bablok regression linear equation showed that postprandial 1,5-AG, GA, and HbA1c levels were well fitted (all *P* > 0.05), and Bland-Altman difference plot showed that 100% of data points for HbA1c_30_ and HbA1c_120_ fell within the limits of agreement; 94.2%, 96.2%, 95.2%, and 95.2% of data points for 1,5-AG_30_, 1,5-AG_120_, GA_30_, and GA_120_ fell within the limits of agreement, respectively.

**Conclusion:**

Agreement analyses indicated good stability of 1,5-AG, GA, and HbA1c levels after the SBMT. HbA1c had an optimal stability, which was superior to that of GA or 1,5-AG.

## 1. Introduction

Fasting blood measurements for the determination of biochemical parameters have long been the norm; however, a shift in this paradigm has increasingly evolved. In a consensus statement issued in 2016, the European Atherosclerosis Society and European Federation of Clinical Chemistry and Laboratory Medicine recommended measurement of nonfasting lipid profile [1], and this has added “nonfasting” measurement of clinical monitoring indicators to the current research hotpot. Extrapolating this to glycemic parameters and assuming 3 meals per day are consumed on average, it can be inferred that the postprandial phase covers 60–70% of a day and represents daily metabolic status more closely [2]. This makes it convenient for both patients and clinical laboratories to collect blood samples without deliberately maintaining a fasting state or a specific time for sample collection. More importantly, levels of nonfasting samples for disease risk assessment were fully affirmed by plenty of research from long-term follow-up studies in large populations [3, 4].

With regard to blood glycemic indicators, glycated hemoglobin A1c (HbA1c) is the gold standard for determining long-term glycemic control. In recent years, glycated albumin (GA) and 1,5-anhydroglucitol (l,5-AG) have been gradually introduced into clinical practice as emerging biomarkers that reflect average glucose levels of the past 2 to 3 and 1 to 2 weeks, respectively. Therefore, assessment of these 3 indicators in the nonfasting state is considered essential in the clinical setting.

Despite this, only a few studies have attempted to validate agreement between baseline and postload levels of these 3 indicators [5–7]. Our previous study indicated that serum 1,5-AG levels slightly increased after a glucose load [8]. Nearly all consistency researches are conducted with the oral glucose tolerance test (OGTT). However, the diagnostic gold standard for diabetes was not routinely used in clinical screening. Ealovega et al. reported that random plasma glucose could be the most common screening method (95%) for diabetes, with OGTT accounting for <1% of screening tests [9]. Furthermore, the blood glucose and insulin profiles after an OGTT and those after daily meals are not exactly interchangeable [10]. Therefore, as monitoring indicators, it is of marked clinical guiding significance to conduct a stability study of postprandial 1,5-AG, GA, and HbA1c after daily meals. The steamed bread meal test (SBMT), with 100 g steamed bread, is used as a simulation to observe the postprandial changes in blood glucose, islet function, and other biochemical indicators [11]. As one of the most popular foods in China, steamed bread accounts for nearly 40% of national wheat consumption [12] and more closely resembles the carbohydrate-based diet of the Chinese population [13, 14].

Previous studies, however, have not explored the trends of 1,5-AG, GA, and HbA1c after a steamed bread meal load, and no study to date has simultaneously compared postprandial agreement of 1,5-AG, GA, and HbA1c. Therefore, based on the previous study, this study was conducted to explore short-term changes in 1,5-AG levels after an SBMT, to evaluate concordance between fasting and nonfasting levels of 1,5-AG, GA, and HbA1c in a representative Chinese population, and to simultaneously compare simultaneous agreement of these 3 indicators to specify the rationale for their clinical application. Consistency analyses were conducted with several methods to evaluate different aspects of central tendency, dispersion, and correlation.

## 2. Materials and Methods

### 2.1. Subjects

The study population (*n* = 104, 76 with diabetes and 28 without diabetes, age range: 47–71 years) was prospectively recruited from the Shanghai Zhabei community between May and July 2016. Each participant underwent a 100 g SBMT, and diabetes was diagnosed on the basis of the 1999 World Health Organization criteria [15]. Individuals with a surgical history of subtotal gastrectomy, severe anemia, chronic liver disease, kidney disease, cystic fibrosis, use of acarbose or glucosidase inhibitors or some traditional Chinese medicines such as *Polygala tenuifolia* and *Senega syrup* therapy, acute infection or other clinically stressful conditions, thyroid disease, cancer, and mental disorders were excluded.

This observational study was conducted in accordance with the principles expounded in the Declaration of Helsinki and was approved by the Ethics Committee of Shanghai Jiao Tong University Affiliated Sixth People's Hospital. All subjects provided written informed consent prior to study participation.

### 2.2. Anthropometric and Biochemical Assessments

We developed a uniformly designed study-specific questionnaire, which included questions on history of past and present illnesses and medication. Standard measurements of body weight, height, waist circumference, and blood pressure were done, and body mass index (BMI) was calculated as BMI = weight (kg)/height^2^ (m^2^). Fasting plasma glucose (PG_0_), 1,5-AG_0_, GA_0_, HbA1c_0_, hemoglobin, albumin, and serum creatinine levels were measured in morning fasting serum samples. Each subject then underwent an SBMT. Approximately 30 and 120 min, respectively, after SBMT, 1,5-AG_30_, 1,5-AG_120_, GA_30_, GA_120_, HbA1c_30_, and HbA1c_120_ levels were determined.

Standard laboratory measurements were performed. Plasma glucose levels were immediately obtained by the glucose oxidase method (Roche Diagnostics GmbH, Mannheim, Germany) using the 7600–120 autoanalyzer (Hitachi, Tokyo, Japan). HbA1c was detected by high-performance liquid chromatography (Variant II hemoglobin analyzer; Bio-Rad, Hercules, CA, USA) with an inter- and intra-assay coefficient of variability (CV) of 0.75–3.39% and 0.55–2.58%, respectively. GA was measured using an enzyme-based assay kit (Lucica GA-L, Asahi Kasei Pharma, Tokyo, Japan) on a 7600–120 autoanalyzer (Hitachi, Tokyo, Japan) with inter- and intra-assay CV of 1.95–4.73% and 1.47–3.30%, respectively. Serum 1,5-AG levels were measured by an enzymatic method (GlycoMark; GlycoMark Inc., New York, NY, USA) on a 7600–120 autoanalyzer (Hitachi, Tokyo, Japan) with inter- and intra-assay CV of 1.54–3.03% and 0.83–2.44%, respectively. Hemoglobin was measured using sodium dodecyl sulfate colorimetry (Sysmex XE-2100 hematology analyzer, Sysmex Corporation, Kobe, Japan), whereas albumin was measured by the bromocresol green method (Kehua Biological Engineering Co. Ltd., Shanghai, China) on a 7600–120 autoanalyzer (Hitachi, Tokyo, Japan). Serum creatinine was measured by the sarcosine oxidase method (Kehua Biological Engineering Co. Ltd., Shanghai, China) on a 7600–120 autoanalyzer (Hitachi, Tokyo, Japan).

### 2.3. Consistency Analysis

Intraclass correlation coefficients (ICCs), as indices of agreement, were calculated as the ratio of individual to total variability. Good consistency was considered when the ICC was higher than 0.75 [16]. The Passing-Bablok regression, a linear regression analysis, was carried out independent of sample distribution and measurement errors. With a linear regression equation, we tested the slope *b* and the intercept *a* to determine the probability that any difference between *b* and 1 and between *a* and 0 arose incidentally. The 95% confidence intervals (CIs) are given [17]. The Bland-Altman difference plot was used to depict differences between the paired postprandial and baseline indicators (baseline levels minus postprandial levels along the *y*-axis against the average of baseline levels and postprandial levels along the *x*-axis). The 95% CIs for the difference ranges (the sample mean difference ± 1.96 standard deviation) reflected the 95% probability range wherein lies the mean difference population parameter [18, 19]. If more than 95% of data points fell within these limits of agreement, a significant systematic difference between the 2 time points for measurement was ruled out.

### 2.4. Statistical Analysis

All statistical analyses were conducted using SPSS 19.0 and MedCalc 12.5.0. Data are presented as mean ± standard deviation. Each variable was examined for a normal distribution, and pair analyses were carried out using paired Student's *t*-test and Wilcoxon signed rank sum test. Intergroup comparisons of skewed data were made using the Kruskal-Wallis test. Spearman correlation analysis was performed to explore the agreement of postprandial levels of the studied indicators. The ICC model, Passing-Bablok regression, and Bland-Altman difference plots were applied to identify the bias of postprandial levels of these indicators. A two-tailed *P* value of <0.05 was considered to indicate statistically significant differences.

## 3. Results

### 3.1. Clinical Characteristics of Study Participants

The 104 participants included 44 men and 60 women, with an age of 62.5 ± 6.1 years. Mean BMI was 24.6 ± 3.2 kg/m^2^. As Table 1 shows, the mean PG_0_, PG_30_, and PG_120_ levels were 7.8 ± 2.2, 9.8 ± 2.9, and 12.1 ± 4.5 mmol/L, respectively.

### 3.2. Acute Changes in 1,5-AG Levels after an SBMT

**Figure 1:**
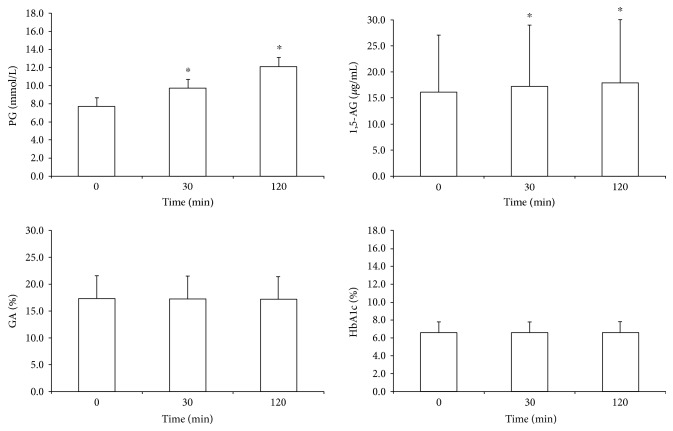
Comparison of baseline and postprandial plasma glucose, 1,5-AG, GA, and HbA1c levels of the entire study population. ^∗^*P* < 0.01 versus fasting levels.

Mean 1,5-AG_0_, 1,5-AG_30_, and 1,5-AG_120_ were 16.1 ± 11.0, 17.2 ± 11.8, and 17.9 ± 12.1 *μ*g/mL, respectively (Figure 1). 1,5-AG increased from 30 to 120 min after a meal, compared with fasting levels (*P* < 0.01). Mean GA_0_, GA_30_, and GA_120_ were 17.3% ± 4.2%, 17.2% ± 4.3%, and 17.2% ± 4.2%, respectively. Mean HbA1c_0_, HbA1c_30_, and HbA1c_120_ were 6.6% ± 1.2% (48.3 ± 12.9 mmol/mol), 6.6% ± 1.2% (48.3 ± 12.9 mmol/mol), and 6.6% ± 1.2% (48.4 ± 12.7 mmol/mol), respectively. No statistically significant differences existed in either GA or HbA1c levels between 30 and 120 min in the SBMT (all *P* > 0.05), and these results were replicated when separately analyzed in patients with diabetes.

### 3.3. Analysis of Agreement in Postprandial and Baseline 1,5-AG, GA, and HbA1c Levels in an SBMT

Spearman correlation analysis showed that postprandial 30 and 120 min levels of the 3 indicators significantly correlated with baseline levels (all *P* < 0.01). ICCs for 1,5-AG_30_, 1,5-AG_120_, GA_30_, GA_120_, HbA1c_30_, and HbA1c_120_ were 0.997, 0.995, 0.989, 0.988, 0.999, and 0.999, respectively.

**Figure 2:**
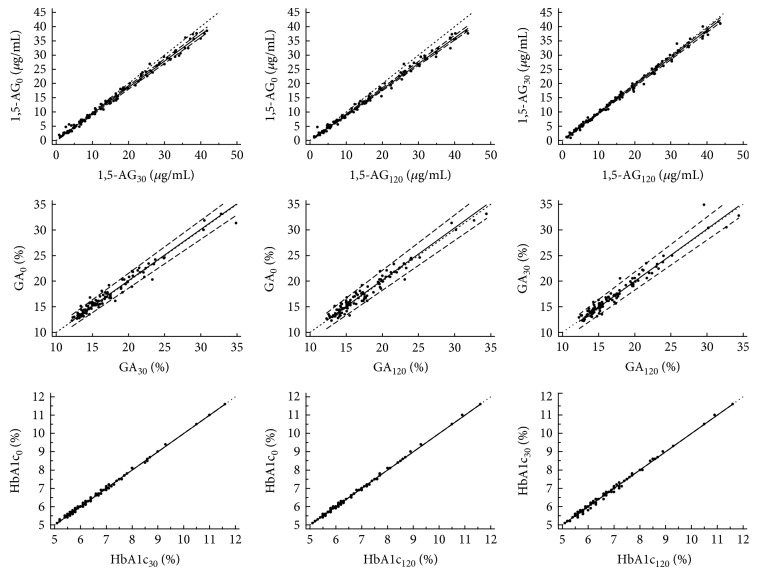
Scatter diagram and linear regression analysis of levels of 1,5-AG, GA, and HbA1c using Passing-Bablok regression analysis.

Passing-Bablok regression analysis showed good agreement between postprandial and baseline HbA1c levels. The linear regression equation was fitted as *y* = 0.000 + 1.000*x* (*P* > 0.05). The fitted linear equations, intercept (95% CI), and slope (95% CI) for postprandial 1,5-AG and GA are shown in Table 2. The linear equations were all well fitted (all *P* > 0.10). There was, further, good agreement between 1,5-AG and GA at 30 and 120 min (*P* > 0.10, Figure 2).

**Figure 3:**
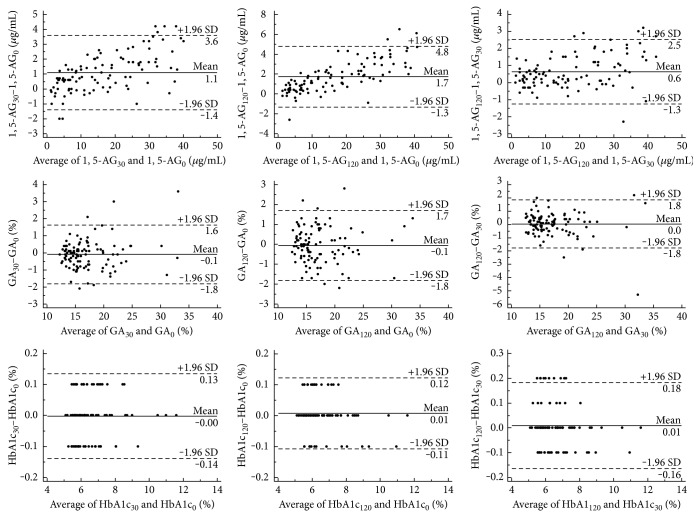
A Bland-Altman difference plot to show the differences between baseline levels and postprandial 1,5-AG, GA, and HbA1c (baseline levels minus postprandial levels along the *y*-axis against the average of baseline levels and postprandial levels along the *x*-axis). Horizontal lines have been drawn at the mean difference (solid line) and at the limits of agreement (for both upper and lower limits of agreement, dashed lines).

Mean differences and 95% CIs between postprandial 1,5-AG levels at every time point and the baseline measurements were 1.1 (−1.4 to 3.6) *μ*g/mL and 1.7 (−1.3 to 4.8) *μ*g/mL. On these graphs, 94.2% and 96.2% of data points for 1,5-AG_30_ and 1,5-AG_120_ fell within the limits of agreement, respectively (Figure 3). Mean differences and 95% CIs between postprandial GA levels at every time point and the baseline GA measurements were −0.1% (−1.8% to 1.6%) and −0.1% (−1.8% to 1.7%). On these graphs, 95.2% and 95.2% of data points for GA_30_ and GA_120_ fell within the limits of agreement, respectively.

Differences of 1,5-AG and GA in the 30 and 120 min after a load were further analyzed by the Bland-Altman difference plot. The difference and 95% distribution range were 0.6 (−1.3 to 2.5) *μ*g/mL, and −0.0% (−1.8% to 1.8%). Outliers of the agreement limits only accounted for 5.8% and 4.8% of the data points, respectively (Figure 3()). The Bland-Altman difference plot showed that 100% of the data points for HbA1c_30_ and HbA1c_120_ fell within limits of agreement and showed significantly better stability than both 1,5-AG and GA.

## 4. Discussion

In the present study, the change trends of 1,5-AG, GA, and HbA1c levels after SBMT in the Chinese population were investigated for the first time. The results showed that similar as the trend after a glucose load, the 1,5-AG levels were slightly elevated after SBMT, whereas levels of GA and HbA1c remained stable. Then, the agreement analyses of the 3 blood glucose-monitoring indicators after SBMT showed good stability, and in the same circumstances, HbA1c showed better stability than both GA and 1,5-AG.

Previous agreement studies of postprandial glycemic indicators were predominantly conducted after an OGTT. The caloric content of 75 g glucose in the OGTT is the same as that of 100 g flour in the SBMT (1255.2 kJ) [20]. However, glucose in the OGTT is in monosaccharide form and is absorbed directly from the small intestine, whereas the SBMT provides polysaccharides in flour that is digested in the small intestine into mono- or disaccharides. Carbohydrates are digested and absorbed at variable rates in different patients, with resultant differences in the insulin release and glucose profiles [21]. The steamed bread meal, which is closer to the daily consumption patterns of patients, could better reflect the impact of meals on glycemic indicators. Further, the SBMT is the most common load evaluation test for diabetes screening in the Chinese population. Therefore, this study selected the SBMT to evaluate the stability of nonfasting measurements of 3 glycemic indicators.

With the HbA1c, a standard glycemic indicator, agreement results after the SBMT were consistent with results reported from previous studies with the OGTT [5, 22] and showed excellent stability. For GA, a study in Taiwan involving 12 individuals without diabetes mellitus who underwent 75 g OGTT, through pair analysis, showed that GA levels were similar in fasting and postprandial samples [7] and concurs with the findings of the present study.

The 1,5-AG accurately and rapidly reflects glycemic control across 1 to 2 weeks [23–27]. Previous research has indicated the superiority of the 1,5-AG over GA and HbA1c for evaluating postprandial blood glucose variability [28]. Few studies, to date, exist on the postprandial agreement of the 1,5-AG. A Japanese study [6] of 77 healthy men showed that there were significant increases at 90, 120, and 180 min after the OGTT. Our previous study involving 681 with differing degrees of glucose tolerance suggested that serum 1,5-AG levels slightly increased rather than decreased after a glucose load [8], which were similar to the results of the present study. A drastic efflux of 1,5-AG into the blood that was induced by acute hyperglycemia may provide a partial explanation to some extent [29, 30].

This study was further compared with the stabilities after an SBMT and found the HbA1c to be the best indicator with 100% points falling within the boundaries of consistency and corresponding value of GA and 1,5-AG of only about 95%. In addition to the stability of the indicators themselves, another possible reason might lie in the current routine clinical assessment methods. HbA1c was measured using high-pressure liquid chromatography, and both 1,5-AG and GA were measured using enzymatic assays. High-pressure liquid chromatography is not influenced by the protein concentration of the sample or the presence of glucose [31]. The study reported by Luconi et al. [32] included twenty-seven patients with type 2 diabetes mellitus who have changed their treatment measures, and they found that variation of HbA1c might represent a better short-time predictor of therapeutic response than GA. This result might be due to the lack of standardization of laboratory methods for GA. On the contrary, the enzymatic method is relatively more sensitive, and results may be influenced by sample parameters, such as hemolysis or lipemia, that may also affect the stability of nonfasting detection of 1,5-AG and GA.

There are some limitations to this study. First, this study with its small sample did not include patients with severe hyperglycemia. Second, this study was performed among a preselected high-risk population, which might considerably affect the sensitivity and specificity of the test. Third, glycemic indicators were evaluated at only 2 time points after the SBMT. Data from more time points in a larger and different glucose tolerance status population could provide a more complete picture of variations in these indicators after a glucose load.

In conclusion, using several consistency analytical methods, this study utilized steamed bread as a simulation of daily postprandial status. The results showed that the levels of 1,5-AG were slightly elevated after the SBMT, whereas those of GA and HbA1c remained stable, similar as the trend after a glucose load. Agreement analyses of the 3 glycemic indicators after the SBMT showed good stability and indicated the superiority of the HbA1c in terms of stability, better than GA and 1,5-AG. These results provide a basis for the application of these 3 glycemic indicators' nonfasting measurement in clinical practice. Finally, the present study provided a better understanding of the role of 1,5-AG in metabolism and supported further studies of its transport. Because the mechanism for this change remains unclear, further studies in different 1,5-AG transport models are needed to investigate the relevant metabolic pathways.

## Figures and Tables

**Table 1:** Demographic and clinical characteristics of study population.

Variable	Total (*n* = 104)
Men/women	44/60
Age (years)	62.5 ± 6.1
BMI (kg/m^2^)	24.6 ± 3.2
Systolic blood pressure (mmHg)	138.2 ± 15.6
Diastolic blood pressure (mmHg)	79.3 ± 8.9
Waist circumference (cm)	85.8 ± 9.1
PG_0_ (mmol/L)	7.8 ± 2.2
PG_30_ (mmol/L)	9.8 ± 2.9
PG_120_ (mmol/L)	12.1 ± 4.5
Hemoglobin (g/L)	141.7 ± 13.0
Albumin (g/L)	49.6 ± 2.6
Serum creatinine (*μ*mol/L)	63.4 ± 14.7

Data are presented as mean ± standard deviation. BMI: body mass index; PG: plasma glucose.

**Table 2:** Passing-Bablok regression analysis equation of three indicators after the steamed bread meal test and baseline levels.

	Passing-Bablok equation	*a* (95% CI)	*b* (95% CI)	*P*
HbA1c_30_	*y* = 0.000 + 1.000*x*	0.000 (0.000 to 0.000)	1.000 (1.000 to 1.000)	>0.10
HbA1c_120_	*y* = 0.000 + 1.000*x*	0.000 (0.000 to 0.000)	1.000 (1.000 to 1.000)	>0.05
1,5-AG_30_	*y* = 0.024 + 0.929*x*	0.024 (−0.243 to 0.285)	0.929 (0.912 to 0.948)	>0.10
1,5-AG_120_	*y* = –0.108 + 0.905*x*	–0.108 (−0.398 to 0.013)	0.905 (0.890 to 0.922)	>0.10
GA_30_	*y* = 0.100 + 1.000*x*	0.001 (−0.513 to 0.812)	1.000 (0.956 to 1.036)	>0.10
GA_120_	*y* = –0.253 + 1.022*x*	–0.253 (−1.226 to 0.501)	1.022 (0.972 to 1.081)	>0.10

*a*: intercept *a*; *b*: slope *b*; HbA1c: glycated hemoglobin A1c; GA: glycated albumin; 1,5-AG: 1,5-anhydroglucitol; CI: confidence intervals.
